# Effect of 20-ppm ozone and 1% chlorhexidine gels on plaque index and *Streptococcus mutans* counts in the dental plaque in 6–12-year-old children: A randomized, double-blind clinical trial

**DOI:** 10.34172/joddd.2023.40461

**Published:** 2023-11-11

**Authors:** Ziya Ebrahim Adhami, Leila Erfanparast, Zahra Molaei, Javid Sadeghi, Azam Yazdanparas

**Affiliations:** ^1^Department of Pediatric Dentistry, Faculty of Dentistry, Tabriz University of Medical Sciences, Tabriz, Iran; ^2^Department of Microbiology, Faculty of Medicine, Tabriz University of Medical Sciences, Tabriz, Iran

**Keywords:** Chlorhexidine gel, Dental plaque, Ozone gel, *Streptococcus mutans*

## Abstract

**Background.:**

One of the methods to control dental caries is to use ozone. Since it is difficult for children to use mouthwashes, the present study aimed to evaluate 20-ppm zone and 1% chlorhexidine (CHX) gels’ effects on the plaque index and *Streptococcus mutans* counts in 6–12-year-old children.

**Methods.:**

In the present double-blind clinical trial, 165 children, 6–12 years of age, referring to the Department of Pediatric Dentistry, Tabriz Faculty of Dentistry, were selected based on inclusion and exclusion criteria and randomly assigned to three groups: ozone gel, CHX gel, and control. The subjects were instructed to place an adequate amount of the gels on all the surfaces of their teeth with one clean finger. The patients and evaluators were blinded to the study groups. The plaque index and *S. mutans* counts in plaque samples were determined before intervention and three weeks after intervention on the buccal surface of the most posterior maxillary tooth (left or right). *S. mutans* counts were determined by culture. STATA software version 14 was used for statistical analyses using Wilcoxon, Kruskal-Wallis, and post hoc Dum tests. Statistical significance was defined at *P*<0.05.

**Results.:**

The 20-ppm ozone and 1% CHX gels significantly decreased dental plaque compared to the control group (*P*<0.05), and their effects were similar (*P*>0.05). These gels significantly decreased the colonies and bacterial counts of *S. mutans* (*P*<0.05).

**Conclusion.:**

The performance of 20-ppm ozone gel in decreasing the dental plaque and *S. mutans* counts was similar to 1% CHX gel.

## Introduction

 Dental caries is caused by the demineralization of the tooth structure by the organic acids produced by the oral bacteria in the dental plaque through the anaerobic metabolism of dietary sugars.^1-5^ *Streptococcus mutans* and *Lactobacillus acidophilus* are found in the cariogenic biofilms and play a significant role in caries.^5,6^ Decreasing the counts of cariogenic bacteria in the dental plaque is one of the measures to prevent the initiation of dental caries and treat it.^7,8^ Significant advances have been made in preventing and treating dental caries during the past century. Chlorhexidine (CHX) is an antimicrobial agent that can inhibit the growth and proliferation of *S. mutans* and potentially prevent dental caries.^9,10^ Using CHX to prevent dental caries is a nonsurgical caries management strategy.^11^ Another way to control the caries process is by using ozone.^12,13^ Using ozone has been successful in managing wound healing, dental caries, oral lichen planus, gingivitis and periodontitis, halitosis, jaw osteonecrosis, postoperative pain, plaque and biofilm, root canals, dentin hypersensitivity, temporomandibular joint disorders, and tooth bleaching procedures.^14-21^ Ozone can be applied in different forms, such as gas, ozone water, and ozone oil.

 Nagayoshi et al^22^ evaluated the effect of ozone water on the survival of oral and plaque microorganisms and reported that it prevented dental plaque aggregation in vitro. Anumula et al^23^ reported that using water containing ozone as a mouthwash resulted in a significant decrease in *S. mutans* counts compared to CHX after 7 and 14 days, suggesting that it could be used as an alternative to CHX.

 It is important to prevent dental caries and improve oral hygiene in 6–12-year-old children who are in the mixed dentition period, with permanent teeth erupting.^14^ Previous studies have suggested different materials to control dental plaque and decrease the load of cariogenic bacteria. One of the methods to manage dental caries is the use of ozone and CHX. In vitro studies have confirmed the role of ozone in controlling dental plaque and decreasing *S. mutans* counts.^24,25^ Ozone water is not very durable, and its preparation requires special tools.^26^ On the other hand, it is difficult to use mouthwashes in this age group. Therefore, in the present study, the gel form of ozone was used in the younger age group, and unlike previous studies, samples were taken from the dental plaque itself. In addition, a previous study used the gel form of ozone by rubbing it on teeth and gingiva to treat gingivitis.^27^ However, no study has evaluated the effect of ozone gel on *S. mutans* in children.

## Methods

 In the present double-blind, randomized clinical trial, sixteen 6–12-year-old children referring to the Department of Pediatric Dentistry, Tabriz Faculty of Dentistry, were evaluated. First, the parents signed an informed consent form. The inclusion criteria consisted of an age range of 6–12 years, systemic health, no known allergy, no antibiotic therapy in the previous three weeks, patients with a high risk of caries (dmft > 3),^28^ and consent to participate in the study. The exclusion criteria consisted of a lack of cooperation in the sampling procedure at the specific time interval, the incidence of allergy symptoms and signs to the materials used in the study, and not observing the instructions provided.

 The sample size was determined at n = 50 in each group using the plaque index value from a study by Indurkar and Verm ^27^ by considering type I error at 0.05 and an 80% study power. To improve the study’s validity, the sample size was increased by 10%, and finally, 55 samples were included in each group. The samples were assigned to three groups: ozone gel, CHX gel, and control, using the random allocation rule. The patients and the examiner were blinded to the study groups. All the subjects were instructed in oral hygiene measures, including toothbrushing and flossing. The subjects in the 20-ppm ozone and 1% CHX gel groups rubbed the gels on their tooth surfaces twice daily for three weeks in addition to routine oral hygiene measures.^29^ In the control group, the subjects rubbed one clean finger twice daily on all the tooth surfaces. The subjects refrained from drinking and eating for half an hour after using the gels.^29^ The educational film was displayed in the parents’ presence and handed to them in person. The intervention in both groups continued for three weeks. Baseline sampling (before intervention) and three weeks after the intervention were carried out in the early morning hours when the children were fasting, and it was recommended that they not brush their teeth or use dental floss before the sampling procedure. After sampling, the subjects brushed their teeth and continued their routine oral hygiene procedures. In all the subjects, the baseline plaque index and *S. mutans* counts were determined in the samples collected from the buccal surface of the most posterior maxillary tooth (right or left side). The samples were taken from all the surfaces of the tooth in question with a toothpick and transferred into a microtube.^29^

 To determine *S. mutans*counts in both the case group and the control group, during the first session, dental plaque samples were taken before determining the plaque index, and each sample was separately transferred to the microbiology laboratory in previously prepared microtubes. In the laboratory, each sample was dissolved in 500 mL of normal saline and evaluated to determine the colony counts in one of the microtubes in each group to determine the proper dilution. In three microtubes, 45 µL of normal saline was placed, and 5 µL from the microtubes containing the dissolved plaque was transferred into the first microtube; 5 µL of the first microtube was transferred into the second, and 5 µL from the second was transferred into the third microtube. This way, three dilutions were prepared in microtubes, and 10 µL from each dilution was separately transferred into MSB (mitis salivarius bacterium) solid agar medium as a selective bacterial culture medium and spread on the plate surface homogeneously. The plates were incubated for 48 hours at 37 °C under 95% nitrogen and 5% carbon dioxide, and the formed colonies were counted. The presence of *S. mutans* was evaluated under a microscope. Of all the plates, the plate with the lowest colony counts was selected as the proper dilution, and the same concentration was used for the rest of the microtubes. After counting, the number of colonies was multiplied by the reverse of the selected dilution. Since 10 µL of the solution was used for culturing on the plate surface for counting the colonies, the achieved numeric value was multiplied by 100 to achieve the colony counts in 1 mL. Therefore, *S. mutans* counts were estimated in CFU/mL.^30^ STATA software version 14 was used for the statistical analyses of the data with Wilcoxon, Kruskal-Wallis, and post hoc Dunn tests. Statistical significance was defined at *P* < 0.05.

## Results

**Figure 1:**
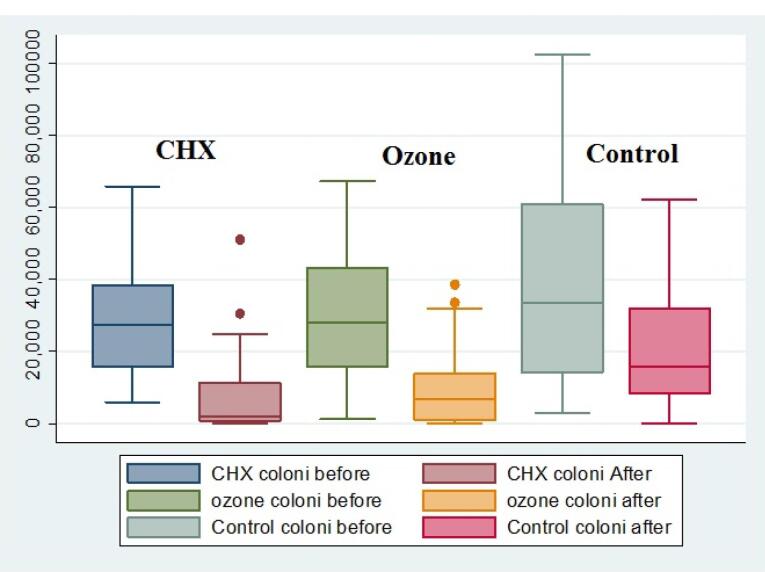
The frequency distributions of colony counts in the study groups before and after the intervention.The means of bacterial counts after intervention: control > ozone > CHX. The rectangle in the middle of each box plot shows the 25, 50, and 75 percentiles, respectively. The 50 percentile is equal to the median of colony counts

 The median bacterial counts (the number of colonies) decreased in all the environments after the intervention; however, this decrease was significant in CHX and ozone environments, with the least median counts in the CHX environment (Figure 1). The results showed that the bacterial counts and plaque sizes significantly decreased in all the groups after the intervention (*P* < 0.05) (Table 1). Table 2 shows no significant differences in bacterial counts (*P* = 0.466) and plaque sizes (*P* = 0.365) between the groups before the intervention. However, there were significant differences in these two variables between the groups after the intervention (*P* = 0.001 for bacterial counts and *P* = 0.003 for plaque sizes). Two-by-two comparisons of the groups showed that the bacterial counts in the CHX and ozone groups were lower than the control group (*P* = 0.001); however, there was no significant difference between the CHX and ozone groups (*P* = 0.380) (Table 3). In addition, plaque sizes in the CHX and ozone groups were significantly less than those in the control group (*P* = 0.001). However, there was no significant difference between the CHX and ozone groups (*P* = 0.430) (Table 3).

**Table 1:** Intragroup comparisons (before and after the intervention) of bacterial counts and plaques between the study groups

**Study groups**		**Before**		**After**		*P* value*
		**Mean**	**SD**	**Mean**	**SD**	
CHX	Bacteria	28750.5	15176	6946.9	10370	0.001
	Plaque	88.32	10.40	30.75	6.70	0.001
Ozone	Bacteria	29469	16781	8930.9	9225	0.001
	Plaque	84.07	16.20	29.75	5.92	0.001
Control	Bacteria	35525.8	23850	20619.6	16735	0.001
	Plaque	86.14	10.76	34.71	6.35	0.001**

*Wilcoxon signed-rank test. **Paired t-test.

**Table 2:** Intergroup comparisons of bacterial counts (colonies) and plaque between the three groups before and after intervention

**Study groups**		**Before** **(Sum ranks)**	*P* value*	**After** **(Sum ranks)**	*P* value*
Bacterial count	Control	4919.5	0.466	6027.5	0.001
	CHX	4351		3451.5	
	Ozone	4424.5		4216	
Plaque size	CHX	4398	0.365	5207	0.003
	Ozone	4972.5		3862.5	
	Control	4324.5		3491.5	

*Kruskal-Wallis test.

**Table 3:** Two-by-two comparisons of the group in terms of bacterial counts and plaque size afterintervention

	**Groups**	**Control**	**CHX**
Bacterial counts after the intervention	CHX	0.001	
	Ozone	0.001	0.380
Plaque size after the intervention	CHX	0.007	
	Ozone	0.001	0.430

*P* values are based on Dunn’s test

## Discussion

 In the present study, 20-ppm ozone and 1% CHX gels were applied to tooth surfaces twice daily for three weeks. The plaque accumulation was similar between the three groups before the intervention; however, there were significant differences between the groups after the intervention, i.e., both gels significantly decreased plaque accumulation compared to the control group. In addition, the effect of CHX and ozone gels on decreasing plaque was similar.

 Consistent with the present study, Bulani et al^31^ reported similar effects of ozone oil and CHX gel on decreasing plaque and gingival indexes as clinical parameters. These researchers showed the antimicrobial and anti-inflammatory effects of ozone on gingival tissue with no complications.

 Gandhi et al^32^ reported no significant differences in PI, GI, PD, CAL, and Pg and Aa counts between the CHX and ozone groups. Since CHX is the gold standard as an anti-plaque and anti-gingivitis agent, ozone-containing olive oil might be used as an auxiliary subgingival irrigation solution in patients with chronic periodontitis. Consistent with the present study, the study above showed similar results concerning the effects of CHX and ozone on decreasing plaque.

 Patel et al^33^ reported that ozone gel with an olive oil base as a sole treatment for periodontitis significantly improved clinical and microbiological parameters over time with no complications. Nagayoshi et al^22^ evaluated the effect of ozone water on the survival of oral microorganisms and dental plaque, concluding that ozone water prevented dental plaque accumulation in vitro.

 Ozone forms oxidized radicals in aqueous environments, disrupting cellular osmotic equilibrium by penetrating the cytoplasmic membrane. It also oxidizes amino acids and nucleic acid, finally lysing the cell.^34^

 In the present study, *S. mutans* counts were evaluated by culturing in the MSB agar medium. Three weeks after the intervention, in all three groups, *S. mutans* colony counts decreased significantly in the CHX and ozone environments, with the least mean colony counts in the CHX group. Evaluation of *S. mutans*counts in the study groups showed that both gels significantly decreased bacterial counts compared to the control group. In addition, the effect of CHX and ozone in decreasing bacterial counts was similar.

 Unlike the present study, Mon et al^35^ reported a better performance of CHX than ozone in decreasing *S. mutans* counts at 15- and 30-day intervals in 10–12-year-old children. These researchers evaluated the salivary samples and reported the lowest debris and Oral Hygiene Index-Simplified Score (OHI-S1) in the ozone water group. Therefore, they recommended ozone-containing water instead of chemical mouthwashes in children.

 When ozone is disintegrated into oxygen, an oxygen-rich environment is created, disrupting the plaque’s natural ecosystem. The cellular enzymatic control system is inhibited because ozone inhibits glycoproteins, glycolipids, and other amino acids, resulting in the cessation of functions and microorganism death.^36^

 Unlike the present study and the studies mentioned in this study, Anumula et al^23^ reported that the effect of ozone-containing water on decreasing *S. mutans* counts was higher than that of CHX; therefore, they suggested continuous use of ozone-containing water as a mouthwash to replace CHX. These researchers evaluated patients with a higher rate of dental caries, with an MS rate > 10^5^ CFU.

 CHX is one of the most commonly used oral antimicrobial agents in different formulations.^37^ CHX exerts lethal effects on bacterial membranes and is active on gram-negative and gram-positive bacteria. It lyses bacterial cell wall and precipitates bacterial cytoplasmic contents. In addition, it destroys bacterial phosphoenolpyruvate and inhibits its metabolic activity.^38^ Moreover, CHX indirectly affects the enzymatic function of dehydrogenase and adenosine triphosphates in the bacterial cell wall, disrupting the cellular membrane.^39^

 Several other studies have shown the effects of ozone on decreasing *S. mutans* counts. For example, Polydoru et al^40^ showed the bactericidal effects of ozone on *S. mutans* after applying it for 80 seconds. In another study, these researchers showed complete inhibition of *S. mutans* growth after applying it for one minute after eight weeks of follow-up.^41^

 According to a study by Safwat et al,^42^ ozone exhibited antimicrobial effects on dental caries in newly erupted permanent molars in Cl I lesions. Estrela et al^43^ reported favorable results concerning the inhibition of *S. mutans* growth using ozone-containing water; however, they also reported limitations in transferring the ozone gas through the used equipment.

 All the above studies have shown the effect of ozone on decreasing *S. mutans* counts; however, using ozone gas has some clinical limitations. Hems et al^44^ reported the limitations of using ozone gas. Using ozone water, too, is associated with some limitations because the O_3_ molecule is unstable, and ozone-containing water should be prepared immediately before being used. Therefore, one of the advantages of the present study was the use of ozone gel, which increases its application, especially in children and adolescents. One of the limitations of the present study was its short follow-up period. Further studies with long-term follow-ups and large sample sizes are required to ensure the positive effect of ozone gel on oral health. In addition, the effect of this gel should also be evaluated on individuals with poor oral hygiene and different caries rates to have a better picture of the antibacterial properties of this gel. In addition, further studies are necessary to determine ozone’s optimal concentration and application time.

## Conclusion

 Considering the effect of ozone gel on decreasing dental plaque and *S. mutans* counts in dental plaque, its use is recommended in 6–12-year-old children.

## Competing Interests

 The authors deny any conflict of interest.

## Ethical Approval

 This study was approved by the Ethics Committee of (Ethics code: IR.TBZMED.REC.1401.304) and registered at the Iranian Registry of Clinical Trials (Identifier: IRCT20220727055562N1; https://www.irct.ir/trial/65219).
